# Food Desert Status of Family Child Care Homes: Relationship to Young Children’s Food Quality

**DOI:** 10.3390/ijerph19116393

**Published:** 2022-05-24

**Authors:** Lucine Francis, Nancy Perrin, Frank C. Curriero, Maureen M. Black, Jerilyn K. Allen

**Affiliations:** 1 Johns Hopkins School of Nursing, Baltimore, MD 21205, USA; nperrin2@jhu.edu (N.P.); jallen1@jhu.edu (J.K.A.); 2 Department of Epidemiology, Johns Hopkins Bloomberg School of Public Health, Baltimore, MD 21205, USA; fcurriero@jhu.edu; 3 Division of Growth and Nutrition, Department of Pediatrics, University of Maryland School of Medicine, Baltimore, MD 21201, USA; mblack@som.umaryland.edu; 4 RTI International, Research Triangle Park, Durham, NC 27709, USA; 5 Division of Internal Medicine, Johns Hopkins School of Medicine, Baltimore, MD 21205, USA; 6 Department of Health, Behavior, and Society, Johns Hopkins School of Public Health, Baltimore, MD 21205, USA

**Keywords:** family child care homes, food deserts, nutrition best practices, beverages, childhood obesity prevention, GIS

## Abstract

Family child care homes (FCCHs) are a favored child care choice for parents of young children in the U.S. Most FCCH providers purchase and prepare foods for the children in their care. Although FCCH providers can receive monetary support from the Child and Adult Care Food Program (CACFP), a federal subsidy program, to purchase nutritious foods, little is known about FCCH providers’ access to nutritious foods, especially among FCCH providers serving children from communities that have been historically disinvested and predominantly Black. This study aims to describe the food desert status of FCCHs in Baltimore City, Maryland, and examine the relationship between food desert status and the quality of foods and beverages purchased and provided to children. A proportionate stratified random sample of 91 FCCH providers by CACFP participation status consented. Geographic information system mapping (GIS) was used to determine the food desert status of each participating FCCH. Participants reported on their access to food and beverages through telephone-based surveys. Nearly three-quarters (66/91) of FCCHs were located in a food desert. FCCH providers working and living in a food desert had lower mean sum scores M (SD) for the quality of beverages provided than FCCH providers outside a food desert (2.53 ± 0.81 vs. 2.92 ± 0.70, *p* = 0.036, respectively). Although the significant difference in scores for beverages provided is small, FCCH providers working in food deserts may need support in providing healthy beverages to the children in their care. More research is needed to understand food purchases among FCCH providers working in neighborhoods situated in food deserts.

## 1. Introduction

Family child care homes (FCCHs) provide care for young children in a home environment outside the child’s home. They are a popular choice for many families due to their affordability, flexibility in hours, proximity to the parental home or workplace, and the feeling of a home away from home [1,2,3]. Children cared for in FCCHs are typically offered breakfast, lunch, and snacks. Unlike center-based child care, FCCH providers often purchase and prepare the food, thus having an opportunity to impact children’s food choices [4]. Early on, a healthy diet consisting of various fruits, vegetables, lean protein, whole grains, and limited sugary beverages can boost immunity and support brain development and oral health in children [5]. A healthy diet can also curb obesity, which currently affects about 14.4 million children and adolescents [6]. To support the purchase of high-quality foods, FCCH providers serving families with limited financial resources can apply to receive monetary support from the Child and Adult Care Food Program (CACFP), a United States Department of Agriculture (USDA) subsidy program [7]. FCCH providers enrolled in the CACFP are more likely to endorse best nutrition practices than non-CACFP FCCHs [8,9,10,11]. Nonetheless, many FCCH providers continue to face challenges concerning rising food costs, coupled with limited reimbursement from CACFP, lack of access to quality supermarkets, and other provider social determinants that may impact the purchase and offering of nutritious foods [12,13,14,15].

Research into the nutrition environment of FCCHs spans from examining nutrition practices through surveys [16,17], observations [18,19], and qualitative interviews [12,20,21,22] to randomized controlled trials of interventions [23,24,25,26,27] to improve the nutrition environment and other proximal outcomes such as business practices and provider health. FCCH-based studies find that providers often report efforts to promote children’s healthy eating despite barriers. For example, although many providers encourage children to try new food, they also report barriers such as food waste and food costs. Implementing well-designed theory-driven multicomponent nutrition interventions for FCCHs significantly improved the children’s diets and their FCCH providers with moderate effect sizes.

Despite the growing body of nutrition-related research in FCCHs, little attention has been paid to the role of the neighborhood food environment of FCCHs in providers’ ability to offer high-quality, nutritious foods. The United States Department of Agriculture and others have used the term food desert to describe areas with limited access to healthy food sources, often measured by distance to a supermarket. This definition also often includes limited individual-level and neighborhood-level resources such as family income, access to a vehicle, and availability of public transportation [28]. Living in a food desert is associated with an increased risk of engaging in obesity-promoting behaviors [29,30], including purchasing unhealthy foods [31]. Racial and ethnic disparities have been associated with food deserts, placing minoritized communities at increased risk for poor diets and cardiometabolic disorders [32,33,34,35,36].

This study aimed to describe and examine whether the quality of foods and beverages offered in FCCHs varied by the food desert status of FCCHs located in Baltimore City, the most populous city in Maryland. Guided by the socio-ecological model of health, which posits that multiple structural factors influence health [37], we hypothesized that FCCHs within a food desert would have a lower quality of foods and beverages offered best practices scores than FCCHs in neighborhoods characterized by closer proximity to supermarkets with high-quality foods, higher family income, and higher vehicle availability.

## 2. Methods

### 2.1. Sample Recruitment and Eligibility Criteria

**Figure 1:**
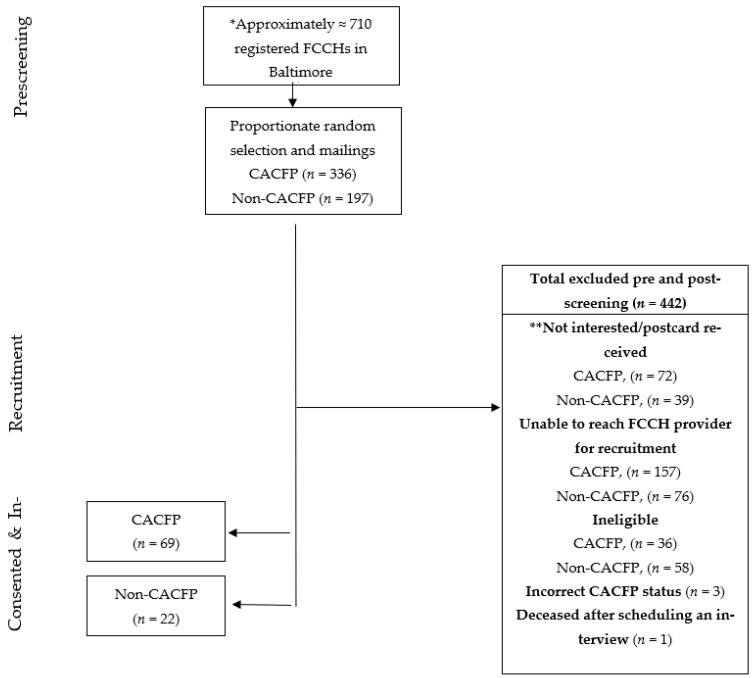
Flowchart describing recruitment efforts by Child and Adult Care Food Program status, * Only 533 mailings were needed to achieve our total sample size of 91. ** Postcard received by study team indicates participant disinterest in being contacted.

We obtained a list of all licensed FCCH providers with their contact information from the Maryland State Department of Education (MSDE), the licensing agency that provides regulatory oversight to child care facilities in Maryland. The list included license numbers, license expiration date, legal names, business addresses, telephone numbers, and the CACFP status of the FCCHs. A proportionate stratified random sample of FCCHs was generated to reflect the 75% CACFP and 25% non-CACFP homes in Baltimore City. We sent recruitment letters in batches of 10–20 weekly or biweekly to the randomly selected providers until the total sample size by CACFP status was achieved. The sample size was set to detect a moderate effect size of 0.30 with a power of 0.80 and 0.05 for the larger study [9]. A pre-stamped return postcard accompanied each recruitment letter for the providers to indicate their disinterest in receiving a telephone call. After two weeks, we contacted providers who did not return the postcard by mail. Providers who were licensed at the time of the call, operated in the city targeted for recruitment, cared for at least one child aged 2–5 years full-time or part-time, provided lunch and snacks, and were able to conduct the 45 min phone survey in English were eligible for the study and consented by phone. Participants were given a USD 25 gift card for a local store for their participation. See Figure 1.

### 2.2. Determining the Food Desert Status of FCCHs

To examine the quality of the neighborhood food environment, we examined the food desert status of each participating FCCH. In partnership with the Baltimore Food Policy Initiative, the Center for Livable Future (CLF) at Johns Hopkins Bloomberg School of Public Health developed a Baltimore City food environment map and defined a food desert as an area where (1) the distance to a supermarket is more than 0.25 miles, (2) the neighborhood median household income is at or below 185% of the Federal Poverty Level, (3) over 40% of households have no vehicle available, and (4) the average Healthy Food Availability Index score for supermarkets and corner stores is low (0–9.5 out of 27), measured using the Nutrition Environment Measurement Survey [38,39,40]. We defined an FCCH as being in a food desert if they met all four criteria or were located within 0.5 miles of a food desert.

We obtained the geographic information systems (GIS) shapefiles for the 2015 Food Desert Map of Baltimore City from the CLF [40]. ArcGIS Desktop version 10.4.1 was used to handle all spatial data, join FCCH addresses to the food desert map, and conduct descriptive analyses [41]. Addresses of the interviewed participants were converted to locations on the food desert map through geocoding [42]. We spatially joined the addresses of the FCCHs to the food desert shapefile to determine the percentage of FCCHs located in food deserts. We created radial buffers around FCCHs outside food deserts to determine which FCCHs were within a 0.5-mile radius of a food desert. We also spatially joined the locations of the FCCHs to each of the four criteria of food deserts to determine which FCCHs fulfilled each criterion of a food desert. Information gathered from the spatial joins (FCCH addresses joined to food desert spatial data in ArcGIS) was used to describe the food desert status of FCCHs.

### 2.3. Study’s FCCH Nutrition Questionnaire

We collected demographic data regarding provider race/ethnicity, height, weight, nutrition training status within the past year, education level, years of child care experience, the number of children in care by age, and racial and ethnic group. We also collected information regarding where providers purchased foods, the average monthly cost of the foods purchased to prepare for the children in their care, and whether they accept child care subsidy vouchers, now known as the child care scholarship, which provides families financial assistance for child care based on their income and resources [43]. All interviews were conducted by one person (LF) between August 2015 and April 2017, with no awareness of the food desert status of the FCCH.

We adapted questions from the Nutrition and Physical Activity Self-Assessment for Child Care, Family Child Care Edition (NAP SACC) to examine the nutrition best practices within the FCCH [44]. A detailed description of how NAP SACC was adapted, including establishing content validity, can be found elsewhere [9]. The tool includes a 4-point Likert scale based on how adequately the child care nutrition standards have been met (1 = barely met, 2 = met, 3 = exceeded, 4 = far exceeded child care standards). The nutritional quality of foods and beverages provided also includes the frequency with which foods and beverages are offered. For example, we assessed the quality of fruits and whether fresh fruits or fruits in 100% fruit juice were provided. Additionally, we considered how frequently fruit was offered (e.g., once/day, twice/day, and so forth). The NAP SACC-selected items were adapted for a structured telephone interview. Several items were adapted to be open-ended questions. For example, the data collector asked, “*How often does your program offer fruit, not including fruit juice?*” Based on the response, the data collector selected either 3 times per week or less, 4 times per week, 1 time per day, or 2 times per day or more. For analyses, each item was dichotomized (best practices reported (score of 3 and 4) vs. not reported (score of 1 and 2)) and summed to create the best practice sum scores for each nutrition best-practice domain. NAP SACC sum scores methods have been used in other studies [9,45]. The NAP SACC yields scores that are the count of the number of best practices in 5 areas: quality and frequency of food offered, based on 11 items ((i.e., fruits, vegetables, meats, grains); beverages offered, based on 4 items (i.e., sugar-sweetened beverages, flavored/unflavored milk, milk type); mealtime environment, composed of 13 items (i.e., role modeling behaviors, respecting satiety, serving meals family style); physical food environment in the FCCH, including 12 items (i.e., nutrition displays, presence of TV during meals); and nutrition-related family engagement, a single item asking about how often information on child nutrition is offered to parents (which can include brochures, tip sheets, or your program’s newsletters, website, or bulletin board and can be offered informally or during meetings or educational sessions with families).

### 2.4. Statistical Analyses

We compared food desert and non-food desert FCCHs on demographics, quality of foods and beverages, mealtime environment score, physical environment score, and parent engagement scores using *t*-tests for continuous variables and chi-square tests for categorical variables. Statistical analyses were conducted using STATA version 16, with *p*  ≤  0.05 as significant [46].

## 3. Results

### 3.1. Demographic Information, Including Food Desert Status

A total of 91 FCCH providers (69 CACFP and 22 non-CAFP) participated in the study for a response rate of 17%. The majority of the FCCH providers were Black or African American (90.1%). The mean (sd) years of child care experience among providers was 18 years (9.5). The providers’ educational backgrounds varied, with the majority having had some advanced training beyond high school (45%). Seventy-eight percent of providers had nutrition training within the past year. Three-quarters of the FCCHs participated in the CACFP. Approximately 72.5% of FCCHs are located in a food desert (in or within 0.5 miles of a food desert). Table 1 summarizes the demographic differences between FCCHs based on food desert status. There were no statistically significant differences in the characteristics of the FCCH providers for any of the variables, including CACFP participation by food desert status.

### 3.2. Quality of Nutrition Practices by Food Desert Status

Table 2 presents the sum score means of quality of nutrition best practices among the FCCHs by food desert status. In support of our hypothesis, FCCHs located within a food desert had a significantly poorer mean quality of beverages, compared to FCCHs outside a food desert (2.53 vs. 2.92; *p* = 0.036, effect size = 0.52). There were no significant differences in the quality of foods, mealtime environment scores, physical environment scores, or parent engagement by food desert status.

## 4. Discussion

We described the food desert status of each participating FCCH and examined the difference in the quality of the foods and beverages offered to young children aged 2–5 years old. In support of our hypothesis, we found that significantly more FCCHs situated within a food desert do not meet best practices for beverages served than FCCHs outside a food desert. CACFP guidelines recommend that children two years and above are offered non-fat, skim, or 1% unflavored milk and no more than 4–6 ounces of 100% fruit juice per day [47]. FCCH providers within food deserts compared to providers outside a food desert significantly differed in their endorsement of sugar-sweetened beverages (i.e., Kool-Aid, fruit drinks, and sweet tea), 100% juices more than once per day, and 2% or whole milk instead of the recommended non-fat skim or 1% milk and flavored milk. Drinking sugary drinks is among the most common obesogenic behaviors in child care [48].

Not supporting our hypothesis, there were no significant differences in the quality of the food served according to food desert status. A potential explanation for this insignificant finding can be due to FCCH providers’ possible reliance on other food sources outside of their neighborhoods or the possibility of food havens—healthy food retail within food deserts. Wholesale warehouses such as Costco were the second leading source of food purchases; providers were likely able to obtain quality foods outside of their neighborhoods despite working and living within a food desert. Another possibility is the emerging evidence that food swamps, which focus on access to unhealthy food options from fast food restaurants and convenience stores, may better predict the quality of consumer food purchases, diet, and risk for obesity compared to food deserts [30,34,49].

Nearly three-quarters of the study’s FCCHs were located in or within half a mile of a food desert, indicating that most FCCH providers lived and worked in neighborhoods with limited access to quality supermarkets. In addition, the neighborhoods were characterized by households with income at the poverty level and no personal vehicle available. The FCCHs outside of food deserts did not differ in demographic characteristics.

We identified only one study that examined the role of the local neighborhood food environment of FCCHs [50]. In that study, conducted in urban and rural areas of Mississippi, the distance to a supermarket and the density of food offering establishments within a five-mile radius of an FCCH were used to define the food environment of FCCHs. The study found that adherence to best nutrition practices among urban FCCH providers differed by their proximity to supermarkets versus small grocery stores and convenience stores. Although access to supermarkets favored some best practices, the study also documented inconsistencies. The recommendation was that healthful food outlets be located close to FCCHs to facilitate access to healthy food and beverages for young children in FCCHs [50].

Several limitations and strengths are present in this study. Self-reporting and fear of reporting poor practices may introduce social desirability biases. To minimize biases, we emphasized to participants that all data would be de-identified and reported in aggregate and that the licensing agency was not part of the study team. The CACFP has updated its meal pattern guidelines [47]. Additional follow-up studies are needed to examine the implications of the revised meal guidelines in FCCHs.

While the concept of food deserts is widely used in the literature, it is important to note that it does not consider the intentional discriminatory practices in reducing access to quality foods and economic opportunities in largely minoritized communities. Food apartheid is a new and emerging concept used to address the limitations of a food desert; however, its conceptual meaning and the ways to measure it are less well-known [51].

Food desert status was uniquely defined for neighborhoods in Baltimore City, Maryland, and may not apply to FCCHs in other locations. However, FCCHs are increasingly common sources of care for young children. The findings may apply to other cities comparable in size to Baltimore City with similar demographics, adverse social determinants of health, and a historical legacy of racism, redlining, and disinvestment. For example, Milwaukee, Wisconsin, and Detroit, Michigan, are mid-sized cities where large populations of people of color reside in communities with poor food access, low-quality supermarkets, and neighborhood poverty. These conditions can affect the quality of foods purchased, consumed, and, ultimately, the cardiometabolic health of the population [52,53,54,55]. Replication studies are needed to determine how neighborhood food access relates to the food environment of FCCHs located within neighborhoods that are low-income.

The study had a limited sample size, which reduces its statistical power. With the current research methods examining the role of neighborhood-level factors on individual behavior cross-sectionally, we cannot indicate any causal relationships.

Our study is one of few that examines the relationship between the neighborhood food environment and nutrition practices within FCCHs. Our findings indicate that access to food outlets is associated with higher-quality beverages. Serving healthy beverages can reduce the risk of obesity and promote health in young children. Consistent with recommendations reported by Braun and colleagues in Mississippi [50], our findings suggest that additional research is needed to describe the local food environments of FCCHs, especially within the context of underserved communities, and advocacy for the provision of high-quality food outlets proximal to FCCHs.

## 5. Conclusions

FCCH providers are an essential child care workforce for families in the US and are uniquely positioned to support the health of the children they serve. Despite FCCHs’ popularity, they have unique challenges. In Baltimore City, Maryland, most FCCH providers live and work in food deserts, which may affect their ability to purchase and provide healthy beverages. Providing healthy beverages to children can help to minimize the amount of calories consumed, support oral health, and prevent obesity. More research is needed to understand some of the neighborhood barriers and community assets to healthy food purchases and nutrition practices in the FCCH.

## Figures and Tables

**Table 1:** Demographic and anthropometric information by the food desert status of each family child care provider (*n* = 91).

	Total	Within Food Desert FCCHs	Outside Food Desert FCCHs	
	(*n* = 91)	(*n* = 66)	(*n* = 25)	
*n* (%) or Mean ± SD				*p*-Value
**Years of Education Mean (SD)**	14.5 (1.7)	14.59 (1.77)	14.30 (1.52)	0.47 ^a^
**Educational Status, *n* (%)**				0.88 ^b^
***<High school***	1 (1)	1 (1.5)	0	
***High*** ***school or GED***	32 (35)	22 (33.8)	10 (40.0)	
***SoMe*** ***College***	41 (45)	30 (46.2)	11 (44.0)	
***≥College***	16 (18)	12 (18.5)	4 (16.0)	
**Years of Experience, Mean (SD)**	18 (9.5)	17.5 (9.61)	19.4 (9.09)	0.40 ^a^
**§** **Black/African American, *n* (%)**	82 (90)	22 (88.0)	60 (90.9)	0.68 ^b^
**CACFP Home**				0.98 ^b^
***Yes***	69 (75.8)	50 (75.8)	19 (76.0)	
***No***	22 (24.2)	16 (24.2)	6 (24.0)	
**Accepts child care subsidy vouchers**	77 (84.6)	57 (86.4)	20 (80.0)	0.45 ^b^
**Typical Food Source For Purchase of Foods (select all that apply)**				
**Supermarkets**	78 (85.7%)	56 (84.8%)	22 (88.0%)	0.70 ^b^
**Wholesale Warehouses (i.e., Costco, BJs)**	53 (58.2)	39 (59.1%)	14 (56.0%)	0.79 ^b^
**Farmer’s Markets**	15 (16.5)	11 (16.7%)	4 (16.0%)	0.94 ^b^
**Grocery Stores**	9 (9.9)	8 (12.1%)	1 (4.0%)	0.25 ^b^
**Corner Convenience Stores**	2 (2.2%)	1 (1.5%)	1 (4.0%)	0.47 ^b^
**Average Monthly Food Costs**	$639 (383.12)	$631 (49.64)	$663 (84.45)	0.74 ^a^
**Number of Children Cared for in FCCH, Mean (SD)**	5.77 (2.37)	5.61 (2.16)	6.20 (2.86)	0.29 ^a^

§ Ethnicity not reported due to too few Hispanics and to protect participant confidentiality; ^a^ Independent sample *t*-test for means; ^b^ Pearson’s chi square test for independence (χ^2^); FCCH = family child care home; CACFP = Child and Adult Care Food Program; completion of H.S. or general education diploma = 12 years; some college = 14 years and above: wholesale warehouses (examples: Costco, B.J.s, Super Walmart, Target).

**Table 2:** Quality of nutrition number of best practices means (standard deviation) by food desert status of family child care home.

	Within Food Desert FCCH	Outside Food Desert FCCH	Range (Lowest-Highest)	*p*-Value	Effect Sizes
**Quality of Foods Offered Score (*w*/*o* beverages)**	8.45 (1.76)	8.60 (1.50)	0–11	0.715	0.09
**Quality of Beverages Provided Score**	2.53 (0.81)	2.92 (0.70)	0–4	0.036	0.52
**Mealtime Environment Score**	9.61 (1.53)	9.64 (1.89)	0–10	0.930	0.02
**Physical Environment (what is available, i.e., Nutrition displays, presence of TV during meals) Score**	5.24 (1.24)	5.08 (1.29)	0–12	0.583	−0.12
**Parent Engagement**—**yes**	50 (86.2%)	19 (90.5%)	0–1	0.614	4.3% difference

## Data Availability

Data can be made available upon request.

## References

[1] Laughlin L. (2013). Who’s Minding the Kids? Child Care Arrangements: Spring 2011. Househ. Econ. Stud..

[2] Child Care Aware of America (2017). 2017 REPORT Checking In: A Snapshot of the Child Care Landscape CCAoA’s Annual State Fact Sheets. https://www.childcareaware.org/wp-content/uploads/2017/07/FINAL_SFS_REPORT.pdf.

[3] ChildCare.gov Family Child Care Homes. https://childcare.gov/consumer-education/family-child-care-homes#:~:text=Manyfamilieschoosefamilychild,intheeveningsorweekends.

[4] Km K., Story M., Kaphingst K.M., Story M. (2009). Child care as an untapped setting for obesity prevention: State child care licensing regulations related to nutrition, physical activity, and media use for preschool-aged children in the United States. Prev. Chronic Dis..

[5] Centers for Disease Control and Prevention (2021). Benefits of Healthy Eating. https://www.cdc.gov/nutrition/resources-publications/benefits-of-healthy-eating.html.

[6] Centers for Disease Control and Prevention (2021). Childhood Obesity Facts. https://www.cdc.gov/obesity/data/childhood.html.

[7] United States Department of Agriculture. https://www.fns.usda.gov/cacfp/child-and-adult-care-food-program.

[8] Ritchie L.D., Boyle M., Chandran K., Spector P., Whaley S.E., James P., Samuels S., Hecht K., Crawford P. (2012). Participation in the child and adult care food program is associated with more nutritious foods and beverages in child care. Child Obes..

[9] Francis L., Perrin N., Black M.M., Allen J.K. (2022). Mealtime Environment and Feeding Practices in Urban Family Child Care Homes in the United States. Child Obes..

[10] Erinosho T., Vaughn A., Hales D., Mazzucca S., Gizlice Z., Ward D. (2018). Participation in the Child and Adult Care Food Program Is Associated with Healthier Nutrition Environments at Family Child Care Homes in Mississippi. J. Nutr. Educ. Behav..

[11] Kenney E.L., Poole M.K., Cory H., Cradock A.L. (2020). Impact of changes to the Child and Adult Care Food Program on children’s dietary intake in family child care homes. Public Health Nutr..

[12] Tovar A., Mena N.Z., Risica P., Gorham G., Gans K.M. (2015). Nutrition and Physical Activity Environments of Home-Based Child Care: What Hispanic Providers Have to Say. Child Obes..

[13] Poole M.K., Cradock A.L., Kenney E.L. (2020). Changes in foods served and meal costs in boston family child care homes after one year of implementing the new child and adult care food program nutrition standards. Nutrients.

[14] Jeon L., Kwon K.-A., Choi J.Y. (2018). Family child care providers’ responsiveness toward children: The role of professional support and perceived stress. Child Youth Serv. Rev..

[15] Dobson P., Burney R., Hales D., Vaughn A., Tovar A., Østbye T., Ward D. (2021). Self-Efficacy for Healthy Eating Moderates the Impact of Stress on Diet Quality Among Family Child Care Home Providers. J. Nutr. Educ. Behav..

[16] Misselhorn A., Hendriks S.L. (2017). A systematic review of sub-national food insecurity research in South Africa: Missed opportunities for policy insights. PLoS ONE.

[17] Patel S.M., Sisson S.B., Stephens H.A., Williams B.D., Hoffman L.A., Salvatore A.L. (2021). Family Child Care Providers’ Nutrition Practices and Policies: Happy Healthy Homes. J. Nutr. Educ. Behav..

[18] Tovar A., Vaughn A.E., Fallon M., Hennessy E., Burney R., Østbye T., Ward D.S. (2016). Providers’ response to child eating behaviors: A direct observation study. Appetite.

[19] Williams B.D., Sisson S.B., Stinner E.L., Hetrick H.N., Dunlap M., Graef-Downard J., Eliot K., Finnell K., Salvatore A.L. (2021). Quality of nutrition environments, menus and foods served, and food program achievement in oklahoma family child care homes. Nutrients.

[20] Lindsay A.C., Salkeld J.A., Greaney M.L., Sands F.D. (2015). Latino Family Childcare Providers’ Beliefs, Attitudes, and Practices Related to Promotion of Healthy Behaviors among Preschool Children: A Qualitative Study. J. Obes..

[21] Rosenthal M.S., Crowley A.A., Curry L. (2013). Family Child Care Providers’ Self-perceived Role in Obesity Prevention: Working With Children, Parents, and External Influences. J. Nutr. Educ. Behav..

[22] Vinci D.M., Whitt-Glover M.C., Wirth C.K., Kraus C., Venezia A.P. (2016). Let’s Wiggle with 5-2-1-0: Curriculum Development for Training Childcare Providers to Promote Activity in Childcare Settings. J. Obes..

[23] Østbye T., Mann C.M., Vaughn A.E., Brouwer R.J.N., Neelon S.E.B., Hales D., Bangdiwala S.I., Ward D.S. (2015). The keys to healthy family child care homes intervention: Study design and rationale. Contemp. Clin. Trials..

[24] Ward D.S., Vaughn A.E., Burney R.V., Hales D., Benjamin-Neelon S.E., Tovar A., Østbye T. (2020). Keys to healthy family child care homes: Results from a cluster randomized trial. Prev. Med..

[25] Sisson S.B., Eckart E., Williams B.D., Patel S.M., Kracht C.L., Davis H.A., Ward D.S., Hildebrand D., Stoner J.A., Stinner E. (2022). Family Child Care Home Providers’ Self-Reported Nutrition and Physical Activity Practices, Self-Efficacy, Barriers, and Knowledge: Baseline Findings from Happy Healthy Homes. Public Health Nutr..

[26] Hazard K., Lee D., Ritchie L., Rose R., Rios L.K.D., Plank K., Alkon A. (2021). Development of an online curriculum for California early care and education providers on healthy beverages. BMC Public Health.

[27] Risica P.M., Tovar A., Palomo V., Dionne L., Mena N., Magid K., Ward D.S., Gans K.M. (2019). Improving nutrition and physical activity environments of family child care homes: The rationale, design and study protocol of the “Healthy Start/Comienzos Sanos” cluster randomized trial. BMC Public Health.

[28] Economic Research Service (2021). Food Access Research Atlas. https://www.ers.usda.gov/data-products/food-access-research-atlas/documentation/.

[29] Caspi C.E., Sorensen G., Subramanian S.V., Kawachi I. (2012). The local food environment and diet: A systematic review. Health Place.

[30] Hager E.R., Cockerham A., O’Reilly N., Harrington D., Harding J., Hurley K.M., Black M.M. (2017). Food swamps and food deserts in Baltimore City, MD, USA: Associations with dietary behaviours among urban adolescent girls. Public Health Nutr..

[31] Caspi C.E., Lenk K., Pelletier J.E., Barnes T.L., Harnack L., Erickson D.J., Laska M.N. (2016). Food and beverage purchases in corner stores, gas-marts, pharmacies and dollar stores. Public Health Nutr..

[32] Berkowitz S.A., Karter A.J., Corbie-Smith G., Seligman H.K., Ackroyd S.A., Barnard L.S., Atlas S.J., Wexler D.J. (2018). Food insecurity, food “deserts,” and glycemic control in patients with diabetes: A longitudinal analysis. Diabetes Care.

[33] Walker R.E., Keane C.R., Burke J.G. (2010). Disparities and access to healthy food in the United States: A review of food deserts literature. Health Place.

[34] Stowers K.C., Jiang Q., Atoloye A., Lucan S., Gans K. (2020). Racial differences in perceived food swamp and food desert exposure and disparities in self-reported dietary habits. Int. J. Environ. Res Public Health.

[35] Ghosh-Dastidar B., Cohen D., Hunter G., Zenk S.N., Huang C., Beckman R., Dubowitz T. (2014). Distance to store, food prices, and obesity in urban food deserts. Am. J. Prev. Med..

[36] Suarez J.J., Isakova T., Anderson C.A.M., Boulware L.E., Wolf M., Scialla J.J. (2015). Food Access, Chronic Kidney Disease, and Hypertension in the U.S. Am. J. Prev. Med..

[37] Bronfenbrenner U. (1986). Ecology of the family as a contexto for human development. Dev. Psychol..

[38] Haering S.A., Franco M. (2010). The Baltimore City Food Environment The Johns Hopkins Center for a Livable Future about the Johns Hopkins Center for a Livable Future. http://www.jhsph.edu/clf/PDF_Files/BaltimoreCityFoodEnvironment.pdf.

[39] Glanz K., Sallis J.F., Saelens B.E., Frank L.D. (2007). Nutrition Environment Measures Survey in Stores (NEMS-S). Development and Evaluation. Am. J. Prev. Med..

[40] Buczynski A.B., Freishtat H., Buzogany S. (2015). Mapping Baltimore City’s Food Environment. Johns Hopkins Center for A Livable Future.

[41] ESRI (2011). ArcGIS Desktop: Release 10. https://www.esri.com/en-us/arcgis/products/arcgis-desktop/overview.

[42] Zandbergen P.A. (2014). Ensuring Confidentiality of Geocoded Health Data: Assessing Geographic Masking Strategies for Individual-Level Data. Adv. Med..

[43] Maryland.gov Child Care Scholarship Program. https://earlychildhood.marylandpublicschools.org/child-care-providers/child-care-scholarship-program.

[44] Ward D. Go NAPSACC: Nutrition and Physical Activity Self-Assessment for Child Care. https://gonapsacc.org/uploads/GoNAPSACC_CN_2020_Copyright.pdf.

[45] Bussell K., Francis L., Armstrong B., Kilby S., Black M.M., Hager E.R. (2018). Examining Nutrition and Physical Activity Policies and Practices in Maryland’s Child Care Centers. Child Obes..

[46] StataCorp (2019). Stata Statistical Software: Release 16. https://www.stata.com/.

[47] United States Department of Agriculture (2016). Child and Adult Care Food Program: Meal Pattern Revisions Related to the Healthy, Hunger-Free Kids Act of 2010, Final Rule. https://www.gpo.gov/fdsys/pkg/FR-2016-04-25/pdf/2016-09412.pdf.

[48] Sisson S.B., Krampe M., Anundson K., Castle S. (2016). Obesity prevention and obesogenic behavior interventions in child care: A systematic review. Prev. Med..

[49] Cooksey-Stowers K., Schwartz M.B., Brownell K.D. (2017). Food swamps predict obesity rates better than food deserts in the United States. Int. J. Environ. Res. Public Health.

[50] Braun L.M., Ward D., Hales D., Vaughn A., Erinosho T. (2022). Food Outlet Density, Distance, and Food Quality Offered to Preschool-Aged Children at Family Child Care Homes. J. Nutr. Educ. Behav..

[51] Agyeman J. (2021). How urban planning and housing policy helped to create “food apartheid” in US cities. The Conversation.

[52] LeDoux T.F., Vojnovic I. (2013). Going outside the neighborhood: The shopping patterns and adaptations of disadvantaged consumers living in the lower eastside neighborhoods of Detroit, Michigan. Health Place.

[53] Budzynska K., West P., Savoy-Moore R.T., Lindsey D., Winter M., Newby P.K. (2013). A food desert in Detroit: Associations with food shopping and eating behaviours, dietary intakes and obesity. Public Health Nutr..

[54] Zenk S.N., Schulz A.J., Israel B.A., James S.A., Bao S., Wilson M.L. (2005). Neighborhood racial composition, neighborhood poverty, and the spatial accessibility of supermarkets in metropolitan Detroit. Am. J. Public Health.

[55] Luevano C. (2021). Food Deserts in Milwaukee. ArcGIS Story Maps.

